# Regulation of Retention of FosB Intron 4 by PTB

**DOI:** 10.1371/journal.pone.0000828

**Published:** 2007-09-05

**Authors:** Victor Marinescu, Patricia A. Loomis, Svetlana Ehmann, Mitchell Beales, Judith A. Potashkin

**Affiliations:** Department of Cellular and Molecular Pharmacology, The Chicago Medical School, Rosalind Franklin University of Medicine and Science, North Chicago, Illinois, United States of America; Centre de Regulació Genòmica, Spain

## Abstract

One effect of stressors such as chronic drug administration is that sequence within the terminal exon of the transcription factor FosB is recognized as intronic and removed by alternative splicing. This results in an open-reading-frame shift that produces a translation stop codon and ultimately a truncated protein, termed ΔFosB. *In vitro* splicing assays with control and mutated transcripts generated from a *fosB* mini-gene construct indicated a CU-rich sequence at the 3′ end of intron 4 (I4) plays an important role in regulating fosB pre-mRNA splicing due to its binding of polypyrimidine tract binding protein (PTB). PTB binding to this sequence is dependent upon phosphorylation by protein kinase A and is blocked if the CU-rich sequence is mutated to a U-rich region. When this mutated *fosB* minigene is expressed in HeLa cells, the splicing efficiency of its product is increased compared to wild type. Moreover, transient transfection of PTB-1 in HeLa cells decreased the splicing efficiency of a wild type *fosB* minigene transcript. Depletion of PTB from nuclear extracts facilitated U2AF^65^ binding to wild type sequence *in vitro,* suggesting these proteins function in a dynamic equilibrium to modulate fosB pre-mRNA alternative splicing. These results demonstrate for the first time that phosphorylated PTB promotes intron retention and thereby silences the splicing of fosB I4.

## Introduction

*Fos* and *jun* are immediate early genes that are expressed rapidly and transiently in the nucleus accumbens (NAc) and other regions of the brain in response to a variety of stressors such as substantia nigra lesions, drug withdrawal and electrical stimulation [1], [2], [3], [4]. These genes encode proteins that are members of the activator protein 1 (AP-1) family of transcription factors. The transcription factors form either heterodimers or homodimers and activate expression by binding to AP-1 sites upstream of particular genes, such as those encoding growth factors and neurotransmitters [1]. Products of the *fos* gene family include cFos, Fos-related antigens (referred to as Fra1 and Fra2) and FosB.

While the 45 kDa FosB protein is only transiently expressed in the brain, ΔFosB, a 33–37 kDa splice variant of FosB, accumulates in a region-specific manner as a common response to chronic stimuli [4], [5], [6], [7], [8]. ΔfosB mRNA is produced by removal of intron 4 (I4) within the terminal exon of the fosB pre-mRNA. This regulated splicing event results in translation termination and the production of a truncated protein. Three factors have been identified that contribute to ΔFosB protein accumulation. These include the repeated activation of the *fosB* gene and induction of ΔfosB mRNA [9]; the absence of a C-terminal destabilizing domain in the case of ΔFosB protein, but not for the other members of the Fos family [10]; and the phosphorylation of an N-terminal serine residue on ΔFosB, which is important in inhibiting proteosomal degradation [11].

Sequencing of the human genome resulted in the surprising finding that more than 42% of the genes produce pre-mRNAs that are alternatively spliced [12], [13], [14], [15]. Many of the regulated splicing events occur in neurons and the molecular basis of the neural specificity is only beginning to be understood [16], [17]. Coding sequences of eukaryotic genes (exons) are interspersed by non-coding sequences (introns) that can be removed from the pre-mRNA by splicing in either a constitutive or a regulated manner. The accurate elimination of these non-coding regions requires a conserved 5′ splice site, a branch point sequence, and a polypyrimidine tract that precedes the 3′ splice site. Interestingly, recent research has indicated that elements critical for the regulation of pre-mRNA splicing may also be found both proximal and distal to the traditional splice sites. These sequences are termed enhancer or silencer elements and can be functional within both introns or exons [18]. Whether the sequence acts as an activator or inhibitor of splicing appears to be context-dependent [19]. Two well-studied examples of exon skipping are those of *c-src* and GABA_A_ receptor γ2 subunit pre-mRNAs, which contain the pyrimidine-rich sequences CUCUCU, UUCUCU, UUCCUU and CUUCUUC [20], [21], [22]. PTB binds these sequences and by doing so functions to silence splicing. The sequence CUCCUCUUCC within transcript generated from the human calcitonin/calcitonin gene-related peptide (CT/CGRP) gene also binds PTB but in this case it enhances inclusion of the adjacent exon 4 into the mRNA [23]. A CU-rich sequence similar to these examples is found proximal to the 3′ end of fosB I4. More recently it was reported that PTB can also counteract the splicing inhibitory activity of SRp30c [24].

Along with identifying target genes that are regulated by the Fos family of transcription factors, recent work has focused on deciphering the regulation of FosB isoform expression. In this study we have used an *in vitro* splicing system to identify PTB as a factor that binds the 3′ end of fosB I4 in a PKA phosphorylation-dependent manner. A CU-rich sequence is required for PTB binding and regulates I4 retention *in vitro* as well as *in vivo*. We also show that U2AF^65^, a factor important in the assembly of the splicing machinery, can bind to the 3′ end of fosB I4. Interestingly, reducing the amount of PTB bound to the 3′ end of fosB I4, by either PTB depletion or mutation of the CU-rich PTB binding element, results in increased binding of U2AF^65^ to fosB I4. These findings support a model in which a competition between PTB and U2AF^65^ regulates fosB I4 retention.

## Results

**Figure 1:**
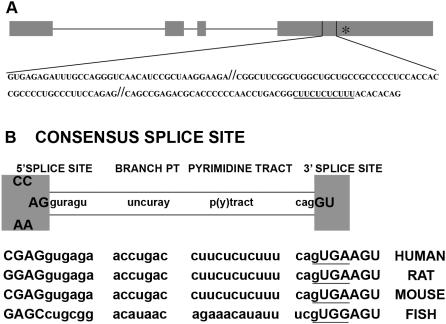
Comparison of fosB I4 sequence among vertebrates. (A) The filled boxes at the top represent fosB exons. The horizontal lines between the boxes represent the three constitutively spliced introns that are removed from the pre-mRNA generated from the gene encoding FosB. The regulated fourth intron is located between the bold-faced vertical lines. Shown below the pre-mRNA is the I4 sequence from the mouse *fos b* gene. The sequence to the left of the first//represents the 5′ I4 transcript; the sequence between the two sets of//represents the middle I4 transcript; the sequence to the right of the second//represents the 3′ I4 transcript. The sequence that is underlined is the PTB binding site. (B) Diagrammatic representation of the consensus splice sites, branch point and pyrimidine tract sequences for vertebrates. The human, rat, mouse and fish fosB I4 splice sites are indicated below. Capital letters represent exonic sequence and lowercase letters represent intronic sequence, n denotes any nucleotide, y for pyrimidines, r for purines and p(y)tract for the polypyrimidine tract, the underlined sequence shows the putative decoy 5′ splice site.

The two splice variants of fosB are products of intron retention (fosB) and intron excision (ΔfosB). A sequence of 140 nt proximal to the 3′ end of the fosB pre-mRNA (Fig. 1A) is removed by splicing to produce ΔfosB mRNA. Visual inspection of the 5′ and 3′ splice sites of the regulated fosB intron, I4, and the upstream and downstream exons 4 and 5 (E4 and E5), revealed that it has been very well conserved between humans, rats and mice (Fig. 1B). However, comparison of the I4 splice site of mammals with that of fish indicated that regulated fosB pre-mRNA splicing has not been evolutionarily conserved among all vertebrates (Fig. 1B). The 3′ splice site of the mammalian fosB I4 is suboptimal when compared to the splice site consensus sequence. One notable change from the consensus sequence is that the first nucleotide of the downstream exon is a U, which deviates from the G present in the consensus sequence. This deviation produces a sequence similar to the consensus 5′ splice site sequence, thereby potentially creating a pseudo 5′ splice site adjacent to the 3′ splice site (Fig. 1B, underlined sequence). The weakness of this splice site is likely to be an important factor in why I4 is usually retained when fosB is expressed.

### Characterization of I4 Binding Proteins

**Figure 2:**
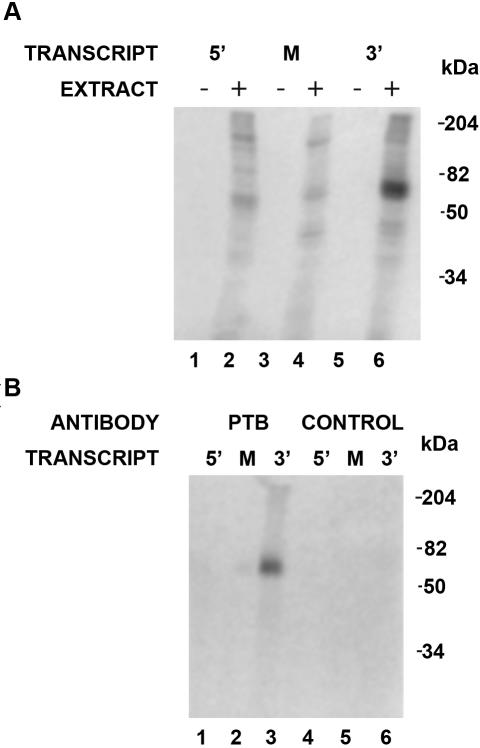
PTB binds to the 3′ end of fosB I4. (A) ^32^P-labeled mini RNA substrates from the 5′, M and 3′ region of I4 were incubated in the absence (−) or presence (+) of HeLa nuclear extract before UV cross-linking, resolved by gel electrophoresis and visualized by autoradiography. (B) ^32^P-labeled mini RNA substrates from the 5′, M and 3′ region of I4 were incubated in the presence of HeLa nuclear extract before UV cross-linking. The samples were then immunoprecipitated with antibody that recognizes PTB or a control antibody that does not recognize splicing factors, resolved by gel electrophoresis and visualized by autoradiography. The 5′, M and 3′ substrates are transcripts from three regions of the intron as defined in the legends of Fig. 1. The position of protein molecular weight markers is indicated on the right in kDa.

With regards to splicing regulation, the binding of *cis*-acting RNA elements becomes very important in dictating whether the spliceosome is recruited to weak splice sites within the pre-mRNA. In order to identify factors that are necessary for the regulated splicing of I4, we created a *fosB* minigene containing exon 4, intron 4 and exon 5 (E4, I4 and E5). The *fosB* sequence was cloned downstream to a T7 polymerase promoter to facilitate production of transcripts. Previous studies showed that serum stimulated HeLa cells produce both FosB and ΔFosB and, therefore, the factors essential for the recognition of I4 are present in these cells [25]. In order to determine which proteins bound to I4 of fosB, radioactively-labeled transcripts were prepared from the 5′, middle and 3′ end of the intron (refer to Fig 1A) and used as substrates in UV cross-linking assays (Fig. 2A). The results from these studies indicated that different subsets of proteins bound to each portion of the intron and that an abundant 59 kDa protein bound the 3′ end of the intron (Fig. 2A, lanes 1–6). Visual inspection of the sequence at the 3′ end of the intron indicated that a putative PTB binding site is present within the polypyrimidine tract at the end of the sequence (Fig. 1A, underlined sequence). PTB is approximately 59 kDa and it binds CUCUCUU sequences in other regulated transcripts [20], [21], [22]. A cross-linking immunoprecipitation assay using an antibody specific for PTB or a control antibody showed that PTB bound to the 3′ end of fosB I4 (Fig. 2B, lane 3).

### PTB Regulates Retention of FosB I4

**Figure 3:**
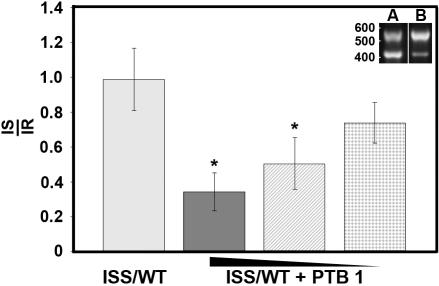
* In vivo* splicing of fosB I4 is regulated by PTB. (A) HeLa cells were transiently co-transfected with ISS/WT and either a control plasmid (bluescript) and/or a construct containing cDNA corresponding to PTB 1. Addition of the control plasmid ensured equal levels of DNA within the transfection assay. The amounts of PTB 1 plasmid used per well in the transfection assays were 1.5 (dark grey box), 1.0 (stripped box) and 0.5 µg (stippled box). The data is representative of single experiment in which all conditions were performed in duplicate. The splicing products of the *fosB* mini-gene transcripts were analyzed by semi-quantitative PCR using primers that detect both the intron retained transcript (IR) and the intron spliced transcript (IS). An example of the PCR products generated from ISS/WT transcripts isolated from HeLa cells co-transfected with either bluescript (A) or PTB 1 (B) is shown in the inset. The data is represented as the ratio of IS to IR mRNA. Bars display the standard error, *p<0.05.

To characterize the role of PTB in the regulation of FosB pre-mRNA splicing, HeLa cells were co-transiently transfected with a plasmid expressing *fosB* (ISS/WT) and either a control plasmid (bluescript, pBS) or a construct containing cDNA corresponding to PTB 1. Forty-eight hours post-transfection RNA was isolated from the transfected cells and cDNA prepared. Ectopically expressed fosB sequence was amplified by semi-quantitative PCR using primers complementary to sequence within the vector and exon 5 (Fig 3). Expression of PTB 1 by transient transfection resulted in a significant decrease in fosB I4 splicing. This effect was gradually diminished when decreasing amounts of the PTB 1 plasmid were used in the transfection assay.

### Analysis of the PTB Binding Site Within the 3′ End of I4

**Figure 4:**
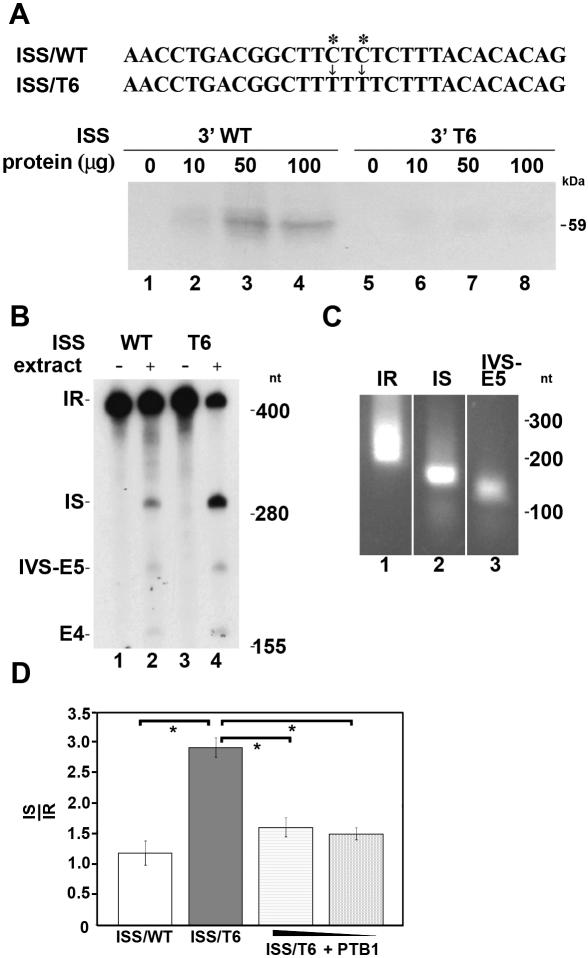
The CT-rich sequence of fosB I4 is required for PTB binding and fosB transcript splicing *in vitro* and *in vivo.* (A) Wild type (ISS/WT) and Mutant (ISS/T6) CT-rich region of fosB I4 sequence. Two nucleotides indicated by asterisks were mutated to disrupt PTB binding (ISS/T6). ^32^P-labeled mini RNA transcripts generated from the 3′ regions of I4 within the ISS/WT and ISS/T6 constructs were incubated in the presence of increasing amounts of HeLa nuclear extract before UV cross-linking. Samples were then resolved by gel electrophoresis and visualized by autoradiography. (B) *In vitro* splicing reactions containing ^32^P-labeled ISS/WT and ISS/T6 fosB pre-mRNA substrate were incubated in HeLa nuclear extract and the resultant products of splicing were resolved by gel electrophoresis and visualized by autoradiography. The size the of RNA markers are shown on the right in nt. IR, IS, IVS-E5 and E4 indicate the intron retained transcript, the intron spliced transcript, the intermediate lariat and exon 4, respectively. (C) RNA products from the gels in (B) were cut out and PCR assays with primers specific for each product were used to amplify the IR, IS and IVS-E5 products. DNA markers are indicated on the right in nt. (D) HeLa cells were transiently co-transfected with ISS/WT (white box) or ISS/T6 mutant *fos B* mini-gene sequence (dark grey box) and either a control plasmid (bluescript) or a construct containing cDNA corresponding to PTB 1. Addition of the control plasmid ensured equal levels of DNA within the transfection assay. The amounts of PTB 1 plasmid used per well in the transfection assays were 1.5 µg (light grey stippled box), and 0.5 µg (dark grey stippled box). The data is representative of single experiment in which all conditions were performed in duplicate. The splicing products of the *fosB* mini-gene transcripts were analyzed by semi-quantitative PCR using primers that detect both the intron retained transcript (IR) and the intron spliced transcript (IS). The data is represented as the ratio of IS to IR mRNA. Bars display the standard error, *p<0.01.

We next sought to repress the expression of PTB within HeLa cells by siRNA technology and determine what effect this would have on the level of splicing of transcripts generated from the *fosB* minigene construct. Although we were able to reduce both the mRNA and protein levels of PTB (data not shown), we were not able to achieve enough of a reduction to have a significant effect on the splicing of transcripts generated from the *fosB* minigene construct. Therefore, we undertook an alternative approach. The *fosB* minigene was modified by site-directed mutagenesis in such a manner that the PTB binding site within the 3′ end of I4 was eliminated, but the pyrimidine-rich nature of the sequence was maintained (Fig. 4A, ISS/T6). This construct was then used for both *in vitro* splicing assays and transient transfection in HeLa cells to further explore the functional effects of the mutation. This approach eliminates the non-target artifacts obtained by siRNA technology. In addition, the global decrease of PTB within cells induced by siRNAs would effect numerous other pre-mRNA reactions and biological processes that are regulated by PTB besides fosB splicing. By contrast, the dominant negative mutation exclusively disrupts the interaction of PTB with fosB I4. Thus, using this approach we are able to obtain a more focused analysis of the role of PTB in the regulated splicing of fosB pre-mRNA.

Transcripts corresponding to the 3′ end of fosB I4 were generated from wild type and mutant constructs and used in cross-linking immunoprecipitation assays. The mutations within the CU-rich region disrupted PTB cross-linking to the 3′ sequence of fosB I4 (Fig. 4A, compare lanes 1–4 to lanes 5–8). The results from these studies indicated that one element critical for PTB binding was within the CUCUU sequence.

In order to assess whether PTB regulates processing of the fosB pre-mRNA, *in vitro* splicing assays were performed with transcripts prepared from constructs containing the ISS/wild type sequence or the ISS/T6 mutation (Fig. 4B). The sizes of the intron retained (IR), intron spliced (IS) and E4 RNAs are 444, 303 and 207 nucleotides (nt), respectively. The products of the splicing assays were confirmed by eluting RNA from the gel and amplifying by PCR using intron- and exon-specific primers (Table 1). Amplification of IR and IS RNAs produced PCR products of 241 and 181 nt, respectively, while the size of the amplified lariat structure was 150 nt (Fig. 4C). The results from the splicing assays showed that when compared to ISS/wild type, the amount of IS increased and IR decreased when the splicing reaction contained ISS/T6 as the source of the transcript (Fig. 4B, lanes 2 and 4). These findings provide support for a regulatory role of PTB in the alternative splicing of the fos B pre-mRNA.

**Table 1:** Oligodeoxynucleotides used in subcloning, PCR, UV cross-linking and mutagenesis studies.

Name	Sequence	Experiment
pG3 forward	5′–CTGAGCTATTCCAGAAGTAGTGAGG-3′	Transfection analysis
pG3 reverse	5′-GCAAGAAGGGAGGGCGAGTTCAGCG-3′	Transfection analysis
WT forward	5′-AGAAACTGATCAGCTTGAAGAGGAAAAG-3′	MiniFosB gene
WT reverse	5′-GTACGAAGGGCTAACAACGG-3′	MiniFosB gene
5′ forward	5′-GCATGCTAATACGACTCACTATAGGGTGAGAGA TTTGCCAGG-3′	UV cross-linking
5′ reverse	5′-TCTTCCTTAGCGGATGTTG-3′	UV cross-linking
Middle forward	5′-GCATGCTAATACGACTCACTATAGGGCGGCTTCGGCTGGCTG-3′	UV cross-linking
Middle reverse	5′-CTGCTCTGGAAGGGCAGG-3′	UV cross-linking
3′ forward	5′-GCATGCTAATACGACTCACTATAGGGCCGAGACGCACCCCCCA-3′	UV cross-linking
3′ reverse	5′-TGTGTGTAAAGAGAGAAGCCGTCAG-3′	UV cross-linking
ISS/T6 forward	5′-CACCCCCCAACCTGACGGCTTTTTTCTTTACACACAGTGAAGTTC-3′	ISS/T6 mutagenesis
ISS/T6 reverse	5′-GAACTTCACTGTGTGTAAAGAAAAAAGCCGTCAGGTTGGGGGGTG-3′	ISS/T6 mutagenesis
Exon 4 forward	5′-AAAAGGCAGAGCTGGAGTCG-3′	PCR of splice products
Exon 5 reverse	5′-GTACGAAGGGCTAACAACGG-3′	PCR of splice products
Intron 4 forward	5′GCATGCTAATACGACTCACTATAGGGCGGCTTCGGCTGGCTG-3′	PCR of splice products

ISS/WT or ISS/T6 reporter *fosB* minigenes were transiently expressed in HeLa cells to further explore the effects on splicing due to the loss of binding of PTB to the 3′ end of I4. Forty-eight hours post-transfection RNA was isolated from the transfected cells, cDNA prepared and then analyzed by semi-quantitative PCR with a single set of primers that detect both I4 retained and spliced transcripts generated from the control and mutagenized *fosB* minigene constructs. The site directed mutagenesis of the PTB binding site in the 3′ end of I4 resulted in a significant enhancement of splicing of the *fosB* minigene transcript (Fig 4D). These results together with those of the PTB binding assays and *in vitro* and *in vivo* splicing assays established a strong correlation between PTB binding to the 3′ end of fosB I4 and intron retention. The decrease in the ratio of IS to IR when excess PTB is present within HeLa cells transfected with ISS/T6 suggests that other PTB binding sites may be able to influence splicing of the *fosB* when the ISS of I4 is mutated. Notably, while the presence of excess PTB for the control condition (ISS/WT) results in an ∼3-fold reduction in the ratio of IS to IR (Fig 3), this reduction for the experimental condition (ISS/T6) is less than 2-fold (Fig 4D). This strongly supports the important role of PTB binding to sequence within the 3′ end of I4 for regulated splicing of fosB pre-mRNA.

### Requirement of PTB Phosphorylation for I4 Interaction

**Figure 5:**
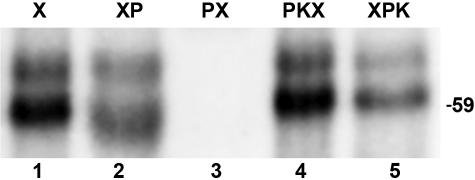
Alkaline phosphatase treatment results in the lack of PTB binding to the 3′ end of fosB I4. ^32^P-labeled mini RNA substrates from the 3′ region of fosB I4 were incubated in the presence of purified his-tagged PTB 1 protein and UV cross-linked (lane 1, X). Alternatively, samples were phosphatase treated either after or before the UV cross-linking step (lanes 2 and 3, XP and PX, respectively) or phosphatase digested and kinase treated before or after cross-linking (lanes 4 and 5, PKX and XPK, respectively). Samples were resolved by gel electrophoresis and visualized by autoradiography. The position of a protein molecular weight marker is indicated on the right in kDa.

Earlier research has shown that phosphorylation can modulate the intracellular transport of PTB, offering a potential means for regulation of its activity in the nucleus [26]. In order to determine whether dephosphorylation of PTB is important for regulating its binding to fosB I4, His-tagged PTB 1 was expressed by transient transfection and isolated from HeLa cells. The isolated protein was cross-linked to the 3′ wild type transcript before or after phosphatase treatment. PTB bound the fosB I4 transcript (Fig. 5, lane 1), and binding was abolished only when phosphatase treatment preceded the cross-linking step (Fig. 5, compare lanes 2 and 3). Previously PTB was shown to be phosphorylated by PKA [26]. In our studies, supplementing the reaction with the catalytic subunit of PKA and ATP, following the phosphatase treatment, was sufficient to rescue PTB binding to the 3′ transcript (Fig. 5B, lane 4). Cross-linking of PTB to this transcript prevents any further disruption of the protein-RNA interaction (Fig. 5, lane 5). The results from these studies suggested that it is not merely the presence of a PTB binding site within the 3′ end of I4 that influences the splicing of fosB pre-mRNA, but rather that this functional interaction is tightly regulated by the PKA-dependent phosphorylation state of PTB.

### Depletion of PTB Results in Enhanced Binding of U2AF^65^ to I4

**Figure 6:**
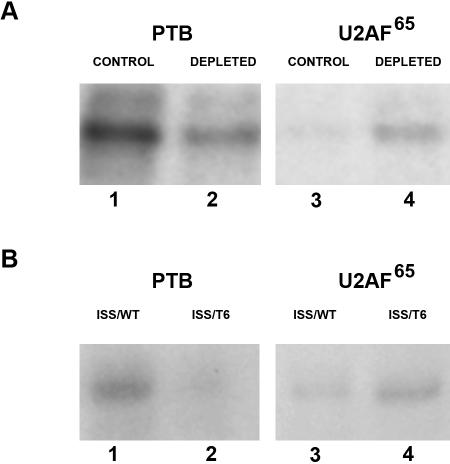
PTB and U2AF^65^ bind to the 3′ end of fosB I4. (A) ^32^P-labeled mini RNA substrate transcribed from the 3′ region of I4 within the ISS/WT construct were incubated in the presence of either control HeLa nuclear extract (lanes 1 and 3) or PTB-depleted HeLa nuclear extract (lanes 2 and 4) before UV cross-linking. The samples were then immunoprecipitated with either an α-PTB antibody (lanes 1 and 2) or an α-U2AF^65^ antibody (lanes 3 and 4). Samples were resolved by electrophoresis and visualized by autoradiography. (B) ^32^P-labeled mini RNA substrates transcribed from the 3′ region of I4 within either the ISS/WT (lanes 1 and 3) or ISS/T6 constructs (lanes 2 and 4) were incubated in the presence of HeLa nuclear extract before UV cross-linking. The samples were then immunoprecipitated with either an α-PTB antibody (lanes 1 and 2) or an α-U2AF^65^ antibody (lanes 3 and 4), resolved by electrophoresis and visualized by autoradiography.

The CU-rich element characterized in this study is located within the pyrimidine-rich tract upstream of the fosB I4 3′ splice site, a region to which the constitutive splicing factor U2AF^65^ binds. One explanation for how PTB silences I4 splicing is that it competes with U2AF^65^ for binding to the intron. To test this hypothesis, we examined whether PTB depletion of the HeLa nuclear extracts would enhance binding of U2AF^65^ to the 3′ wild type sequence of I4. PTB was depleted from the nuclear extracts by incubation with biotin-labeled PTB substrate and streptavidin precipitation as previously described [22]. ^32^P-labeled RNA substrate corresponding to the 3′ region of I4 were incubated in the presence of either control HeLa nuclear extract (Fig. 6A, lanes 1 and 3) or PTB-depleted HeLa nuclear extract (Fig. 6A, lanes 2 and 4). Following UV cross-linking, the nuclear extracts were immunoprecipitated with either an α-PTB antibody (Fig. 6A, lanes 1 and 2) or an α-U2AF^65^ antibody (Fig. 6A, lanes 3 and 4). Samples were resolved by SDS-PAGE and visualized by autoradiography. U2AF^65^ interaction with the 3′ end of I4 was enhanced when the HeLa nuclear extract was first depleted of PTB (Fig. 6A, lanes 3 and 4). Additionally, we analyzed the binding of U2AF^65^ to transcripts which contain the mutagenized PTB binding site within the 3′ end of I4. ^32^P-labeled transcripts corresponding to the 3′ end of fosB I4 generated from ISS/WT (Fig 6B, lanes 1 and 3) and ISS/T6 (Fig 6B, lanes 2 and 4) were incubated in the presence of HeLa nuclear extract before UV cross-linking. Samples were then immunoprecipitated with either an α-PTB antibody (Fig 6B, lanes 1 and 2) or an α-U2AF^65^ antibody (Fig 6B, lanes 3 and 4), resolved by SDS-PAGE, and visualized by autoradiography. The decreased level of PTB interaction with the 3′ end of I4 due to the T6 mutation resulted in a corresponding increase of U2AF^65^ binding to the mutagenized transcript (Figure 6B, lane 2 and 4). In summary, it appears that PTB and U2AF^65^ may compete for binding to the 3′ end of fosB I4 thus providing an additional level of regulation for the splicing of the fosB pre-mRNA.

## Discussion

The brain's ability to adapt in due course to repeated insults can be characterized by the longevity of the response, even after the perturbation has halted. Some examples of such adaptations include learning and memory as well as drug addiction and severe stress [1], [3]. Understanding the molecular basis underlying the stability of these responses has received much attention from the scientific community. The multifaceted process of gene expression is critical to the brain's adaptive response. Therein, considerable research has been focused on the function and regulation of transcription factors including FosB [8], [27], [28], [29].

FosB has been of particular interest since several isoforms, generated by alternative splicing, are differentially expressed in the brain. To date, the expression of ΔFosB is one of the longest-lasting molecular changes produced by chronic drug administration observed in the brain. It is therefore thought to mediate some of the neural and behavioral changes that occur with drug addiction [1], [30]. ΔFosB protein accumulates in the NAc and dorsal striatum of animals chronically treated with cocaine [6]. The truncated protein is produced when a usually retained intron, I4, is deleted from the *fosB* pre-mRNA by alternative splicing. The removal of I4 results in a shift in the open reading frame and produces a translation stop codon [31]. In this study we show for the first time that the binding of PTB to a CU-rich region within the 3′ end of fosB I4 prevents the splicing of I4 and hence the downstream production of mRNA encoding ΔFosB.

### PTB Regulates Intron Retention

In many examples of splicing regulation, the role of PTB is to enhance exon skipping by antagonizing exon definition [22]. Exon definition is the standard mode of recognition of splice sites in vertebrates where genes are composed of relatively short exons interspersed between long introns. In contrast, yeast and other organisms with short introns the usual mode of splice site recognition is by intron definition. The role of PTB in fosB splicing is somewhat unusual in that it is similar to intron definition since the I4 is only 140 nt long. Our data suggest that when PTB binds to fosB pre-mRNA, I4 is retained. Mutating the CU-rich sequence to a U-rich sequence inhibits PTB binding and the mutated fosB RNA was more efficiently spliced both *in vitro* and *in vivo*. This result is in agreement with several studies in which PTB was shown to repress splicing [18], [22], [32], [33], [34]. Because the CU-rich sequence overlaps with the predicted binding site of U2AF^65^, a factor that recognizes the polypyrimidine stretch at the 3′ end of introns, one possible mechanism of PTB function is a simple competition for the binding site [22]. Our finding that depletion of PTB from the HeLa nuclear extract resulted in binding of U2AF^65^ to the wild type sequence supports this mechanism. However, in conjunction with competition between splicing factors, other mechanisms may play a role in the regulation of fosB pre-mRNA splicing by PTB. One such example could be the regulation of propagative binding of PTB along a specific segment of the pre-mRNA [22]. Recent work has suggested that repression of splicing of the SM exon within the α-actinin pre-mRNA may be due the cooperative binding of PTB within the 3′ end of the upstream intron out-competing the binding of U2AF^65^ to the polypyrimidine tract [35]. Examination of the sequence of the *fos* gene using the biocomputational tool EMBL-EBI Splicing Rainbow (http://www.ebi.ac.uk) revealed the presence of three putative PTB binding sites within I4 and exon 5. The alteration of the splice ratios of transcripts containing I4 within HeLa cells co-transfected with ISS/T6 and PTB 1 suggests that under certain conditions additional PTB binding sites within the fos B pre-mRNA may play a role in splicing regulation. The successive binding of PTB may itself be regulatory by interfering with the binding of other splicing factors, and/or functioning to create a scaffold on the pre-mRNA through the molecular interactions between the bound PTB proteins. Interactions between upstream and downstream bound PTB could result in a looping of the RNA, thereby shielding specific sequences from functionally interacting with both splicing enhancing factors and the spliceosome [22]. Results from the ISS/T6 mutation clearly indicate the importance of PTB binding to the 3′ end of I4 for the regulated splicing of fos B pre-mRNA. We are currently examining if interactions between PTB bound at the 3′ end of I4 and additional sites on the fos B pre-mRNA are important for regulated splicing.

While preceding models of PTB function suggest a mechanism of splicing repression by obstruction of splice sites or averting interaction with the spliceosome through looping of the pre-mRNA [22], [33], [34], recent data from the Fas and *c-src* model systems suggest a more complex mechanism [36], [37]. The proposed mechanism involves specific interference with the bridging between U1 small nuclear ribonucleoprotein (snRNP) and U2AF^65^ that is responsible for intron and exon definition. This form of interference cannot be ruled out in the fosB model. A unique aspect in the fosB model that makes it different from the one proposed by Sharma and colleagues [37] is that the I4 3′ splice site is a weak splice site that contains a pseudo 5′ splice site with a putative U1 binding site at the I4/E5 border. If U1 snRNP associated factors indeed bind this site it would most likely block the binding of factors required for the recognition of the 3′ splice site. Further studies are needed to determine if PTB simply obstructs the fosB splice site or perhaps prevents cross-talk between components of the spliceosome and whether factors that bind the 5′ splice site are also involved in the regulation of splicing.

Adding further complexity to the regulation of alternative splicing by PTB is its relationship to brain PTB (nPTB). This splicing factor is a paralog of PTB that is enriched in neurons and a few other tissues [27], [38], [39], [40]. Although these two highly homologous proteins can interact with the same cis-elements on RNAs, nPTB is a significantly weaker silencer of splicing than PTB [41]. Interestingly, during neuronal differentiation PTB levels are decreased via the action of a neuron-specific microRNA which results in a concurrent increase in the level of nPTB [42]. Additionally, siRNA induced knockdown of PTB also induces the expression of nPTB [27], [43]. It is possible that we did not observe significant alterations in the splicing of fosB transcripts in cells in which PTB had been knockdown due to this phenomenon (Marinescu and Potashkin, unpublished). The analysis of the intricate dynamics between these proteins indicates that exon populations possess a graded sensitivity to the repressive effects of the individual PTB paralogs [43]. We have determined that nPTB can bind to the 3′ end of I4 (data not shown). Whether this binding is relevant to the splicing of fosB pre-RNA within the brain is currently unknown but is a focus of ongoing analysis.

The other unique aspect of the function of PTB in the splicing of fosB is that it is an example of intron retention, as opposed to its normal role in exon skipping. Alternative splicing is a common mechanism for producing multiple products from a single gene. Intron retention, however, is a relatively rare form of regulated splicing in vertebrates [44], [45], though it is more prominent in viral splicing. Interestingly, a recent study identified 88 examples in which a retained intron produced a truncated protein in humans and in 40% of these cases the premature stop codon was present in the last exon, similar to fosB [44]. In vertebrates, one well-studied example of intron retention that is similar to fosB is bovine growth hormone (bGH) [46]. The regulated intron of bGH also has suboptimal splice sites. The splicing of bGH is regulated by two constitutive splicing factors; SF2/ASF stimulates removal of the final bGH intron and hnRNP A1 inhibits its splicing [47]. Our results showing that PTB, formerly referred to as hnRNP I, regulates fosB splicing are consistent with the idea that suboptimal splice sites are sensitive to fluctuations in regulatory splicing factors. To our knowledge this is the first example of PTB playing a role in intron retention.

### Phosphorylation of PTB is Critical for RNA Binding

The key factor important for recognition of the suboptimal fosB I4 splice sites may simply be the amount and/or type of PTB present in the nucleus where regulation is occurring. Our novel finding that only phosphorylated PTB binds to the 3′ intron sequence can be interpreted as evidence that phosphorylation of this splicing factor is important for regulation. Recently, it has been shown that PTB shuttles between the nucleus and cytoplasm, and, that this movement is dependent on the phosphorylation of PTB [26]. Specifically, phosphorylation on Ser16 can sequester PTB in the cytoplasmic compartment. These studies did not rule out the possibility of PTB being phosphorylated on additional sites. Ser16 of PTB is not within the RNA binding domains of PTB. This would suggest that the effects of PTB phosphorylation observed in our studies may be due to phosphorylation by PKA on additional sites that are important for RNA/protein interactions. We are currently performing mutational analysis of the PKA sites within PTB to determine which site(s) are critical for regulation of fosB splicing. Additionally, further studies to characterize the distribution of phosphorylated PTB under conditions in which the splicing of fosB is regulated are being carried out in order to fully understand the role PTB in this process.

## Materials and Methods

### Cloning and mutagenesis

A minigene construct containing the last 156 nucleotides (nt) of exon 4 (E4), I4 and the first 167 nt of exon 5 (E5) of *Mus musculus fosB* was created for use as a template for splicing and binding assays (a gift of Dr. Y. Nakabeppu, Kyushu University, Japan). The *fosB* minigene was amplified by PCR and subcloned into both the pGEM-T Easy plasmid (Promega, Madison, WI), adjacent to the T7 promoter to create pEZ_ISS/WT, and a modified pGL3 plasmid (Promega), adjacent to a SV40 promoter to create ISS/WT for transfection studies.

pEZ ISS/WT was used as a template to generate the mutant pEZ_ ISS/T6. A set of complementary oligonucleotides containing the desired mutations was synthesized (Table 1) and used as primers for site-directed mutagenesis using a Quickchange kit (Stratagene, La Jolla, CA). Nucleotides 128 and 130 within the alternatively spliced intron were changed from C to T (C_128_T_129_C_130_→TTT), in order to convert a putative PTB-binding site into a pyrimidine-rich sequence not recognized by PTB. The mutated *fosB* minigene was amplified by PCR using 50 ng of pEZ_ISS/WT construct, 125 ng of sense and anti-sense primers, 0.2 mM each of dTTP, dATP, dCTP and dGTP and 2.5 U of PfuTurbo DNA polymerase (Stratagene) in a buffer containing 10 mM KCl, 10 mM (NH_4_)_2_SO_4_, 20 mM Tris-HCl (pH 8.8), 2 mM MgSO_4_, 0.1% Triton X-100 and 0.1 mg/ml bovine serum albumin (BSA). Eight percent dimethylsulphoxide (DMSO) was added to the reaction to increase the specific binding of the primers. The cycling parameters used were as follows: denature at 95°C for 30 sec, 20–30 cycles at 95°C for 30 sec, 55°C for 1 min, and 68°C for 8 min. Reactions were cooled to 37°C and incubated with *Dpn* I (10 U/µl) for 1 hr in order to digest the parental template so that only mutated DNA remains. Constructs were sequenced to confirm that the mutation. The pEZ_ISS/T6 sequence was also subcloned into a modified pGL3 plasmid (Promega), adjacent to a SV40 promoter to create ISS/T6 for transfection studies.

### Nuclear extract preparation, transcription and *in vitro* splicing assays

Soluble extracts were prepared from nuclei isolated from 11 liters of HeLa cells grown in spinner cultures using a modification of the Dignam method [48], [49]. Templates for splicing substrates were generated by digestion of pEZ_ISS/WT with *Nar* I. Labeled RNA was synthesized from linear plasmid in a 50 µl reaction containing 2 µg of DNA, 0.4 mM each of ATP and GTP, 0.1 mM UTP and CTP, 2.5 µl of [^32^P]-UTP and [^32^P]-CTP (ICN), 0.5 mM diguanosine triphosphate and 2 µl of 20 U/µl T7 RNA polymerase Plus (Ambion, Austin, TX). Reactions were incubated at 37°C for 1 hr and then digested with DNase I for 15 min at 37°C. Transcripts were purified on 4% denaturing polyacrylamide gels, cut from the gel and eluted on a rotating platform in RNA elution buffer containing 2 M ammonium acetate, 1% SDS and 25 µg/ml yeast tRNA at 37°C overnight. RNA substrate was then extracted with phenol:chloroform and ethanol precipitated. *In vitro* splicing assays were done as previously described using 11 µl of HeLa extract (2.6 µg/µl) in a total reaction of 25 µl [50]. Spliced products were separated on 4% denaturing polyacrylamide gels.

Templates for intronic transcripts were prepared by PCR using oligonucleotides listed in Table 1. All forward primers contained a T7 RNA polymerase promoter. Each PCR reaction (20 µl) contained 0.88 ng of pEZ_ISS/WT template, 20 pmol of each primer, 0.25 µl Taq polymerase (5U/µl, Promega), 2 µl of 10× Taq buffer (Promega) and 1 µl of dNTP mix containing 10 mM dATP, dCTP, dGTP and dTTP. The parameters were as follows for 35 cycles: denaturation at 94°C for 15 sec, annealing for 15 sec at variable temperatures dependent upon the primers (5′ intron template primers: 53.3°C, middle intron template primers: 73.1°C, and 3′ intron template primers: 67.9°C), and extension: 72°C for 30 sec. PCR reactions were then extracted with phenol:chloroform and ethanol precipitated.

Intronic transcripts were prepared using PCR generated templates. Transcription reactions (50 µl) contained 2 µg of template, 2.5 µl of 200 U/µl T7 RNA polymerase Plus (Ambion), transcription buffer (Ambion), 16 mM MgCl_2_, 10 mM dithiothreitol (DTT), 4 mM each of ATP, CTP, GTP and UTP and 40 units of RNasin (Promega). Reactions were incubated at 37°C for 1–2 hr and then RQ DNAse (Promega) was added and incubated for an additional 15 min. RNA was phenol:chloroform extracted, ethanol precipitated and resuspended in water. RNA was quantified by measuring absorbance at 260 nm.

Radioactively-labeled intron transcripts were synthesized in 50 µl reactions containing 1 µg of PCR generated template, 0.5 mM each of ATP and GTP, 0.1 mM UTP and CTP, 2.5 µl of [^32^P]-UTP and [^32^P]-CTP (ICN) and 2 µl of 200 U/µl T7 RNA polymerase Plus (Ambion). Reactions were incubated at 37°C for 1 hr and then digested with DNase I for 15 min at 37°C. Transcripts were purified on 10% denaturing polyacrylamide gels and then processed as described for the splicing substrates.

### Identification of fosB splicing products

Products of splicing reactions using transcripts generated from the *fosB* minigene constructs were cut out of the gel and eluted overnight in Elution Buffer. RNA was ethanol precipitated and reverse transcribed into cDNA (SuperScript II RT, Stratagene) in a 20 µl reaction containing 50 ng random primers, 5 µl of RNA and 1 µl dNTP mix (10 mM each). The mixture was heated to 65°C for 5 min and 4 µl of 5× first strand buffer, 2 µl of 0.1 M DTT and 1 µl RNaseOUT (40 units/µl) were added to the reaction. SuperScript II RT (1 µl, 200 U/µl, Invitrogen, Carlsbad, CA) was added to the reaction and incubated at 42°C for 50 minutes. The reaction was inactivated by incubation for 15 min at 70°C. PCR reactions (50 µl) contained 5 µl 10× PCR buffer (200 mM Tris-HCl pH 8.4, 500 mM KCl), 1.5 µl 50 mM MgCl_2_, 10 mM dNTP mix, 1 µl forward primer (10 µM), 1 µl reverse primer (10 µM), 0.4 µl Taq DNA polymerase (5U/µl) and 2 µl cDNA. When intron 4 spliced (IS) and intron 4 retained (IR) RNAs were amplified, a set of E4- and E5-specific primers were used (Table 1). An I4-specific forward primer and an E5-specific reverse primer were used to amplify lariat structures (Table 1). Samples underwent 35 cycles of amplification using the following cycling parameters: 15 sec at 94°C, 15 sec at 60°C or 66°C depending on the primers used, and 30 sec at 68°C. The PCR products were separated on 2% agarose gels.

### UV cross-linking assays

UV cross-linking assays were done as previously described [51]. Assays contained 50,000 cpm of [^32^P]UTP labeled 5′, middle and 3′ substrates in a 10 µl reaction containing 5 µl of HeLa extract (2.6 µg/µl). UV cross-linking reactions were assembled on ice, incubated for 3 min at 30°C and then irradiated in open 1.5 ml centrifuge tubes with 1.2 J, 6.5 cm from a UV light source using a Stratalinker (Stratagene). After exposure to UV light, the reactions were incubated with RNase A (1 mg/ml, Sigma-Aldrich, Saint Louis, MO) for 20 min at 30°C. Reactions were terminated by the addition of protein loading buffer and heating at 95°C for 5 min before loading on 10% SDS polyacrylamide gels. The gels were fixed with 45% methanol and 9% acetic acid before drying and exposing to film.

### UV cross-linking immunoprecipitations

Uniformly-labeled [^32^P]UTP 5′, middle or 3′ RNA substrates were UV cross-linked and RNase A treated as described above. A 10 µl aliquot of a 1:50 dilution of the anti-PTB antibody, Bb7 [52] (a gift of Dr. D.L. Black, UCLA), anti-U2AF^65^ (Santa Cruz Biotechnology, Santa Cruz, CA) or nonspecific antibody (anti-RIF 2C1, a gift of Dr. K. Beaman, RFUMS) was added to each reaction and incubated at 4°C overnight. The following day 20 µl of Protein A agarose (Roche Diagnostics, Mannheim, Germany) was added to each reaction prior to incubation at room temperature for 1 hr. NET buffer (1 ml) containing 50 mM TrisHCl pH 7.5, 150 mM NaCl, 5 mM EDTA and 0.5% NP40 was added to each sample. Precipitates were collected by centrifugation at 600 × g at 4°C for 30 sec. The samples were washed four times in NET buffer. After the final wash the precipitate was resuspended in loading buffer, boiled for 3 min and then loaded on 10% SDS polyacrylamide gels. The gels were fixed with 45% methanol and 9% acetic acid before drying and exposing to film.

### Cell Culture and Transient expression assays

HeLa cells were cultured in DMEM supplemented with 10% fetal bovine serum. His-tagged PTB 1 (human PTB 1, a gift from Dr. J. Patton, Vanderbilt University), was transiently transfected into HeLa cells plated at ∼75% confluency in a 100 mm culture dish using the Lipofectamine reagent (Invitrogen) according to the manufacturer's protocol. Twenty four hours after transfection the cells were trypsinized and replated into two 100 mm culture dishes and cell lysates were collected the following day for protein isolation as described below. Slight modifications of the methods described above were also used to transiently transfect pBS (bluescript); ISS/WT; ISS/T6; and PTB 1 constructs into HeLa cells for RNA analysis. Cells were plated into 12 well culture plates (∼35% confluency) ∼4 hr prior to transfection. Each experimental condition was performed in duplicate. Additionally, each experimental protocol was performed at least three times. Forty-eight hr post-transfection RNA was isolated using the Trizol method, DNase-treated as described above, and cDNA generated. The cDNA was amplified by PCR using a forward primer designed to be complementary to sequence within the pGL3 vector, therefore, only ectopically expressed fosB sequence was amplified. The reverse primer was located in exon 5, thus allowing the amplification of both fosB and ΔfosB cDNA in a PCR reaction with a single set of primers. Control PCR reactions with primers specific for GAPDH were also performed. The amplified products were resolved on 2.5% agarose gels then imaged and quantitated using Kodak 1D Image Analysis Software (Kodak, Rochester, NY).

### PTB protein isolation

The Ni-NTA Purification System (Invitrogen) was used for the purification of polyhistidine-containing recombinant proteins. Cells were suspended in 1.5 ml native binding buffer (250 mM NaH_2_PO_4_ pH 8.0/2.5 M NaCl) supplemented with a protease inhibitor cocktail (Roche Diagnostics) and a phosphatase inhibitor cocktail (1∶100, Sigma-Aldrich). Cells were lysed by a freeze-thaw cycle and the DNA sheared by passing the resulting cell lysate through an 18-gauge needle followed by centrifuged at 3,000×g for 15 min. Ni-NTA columns were prepared by washing 75 µl of Ni-NTA agarose three times with native binding buffer. Lysate (1.5 ml) was then incubated on the column for 30 min, followed by four washes with native wash buffer (native binding buffer supplemented with 20 mM imidazole, pH 8.0). The supernatant was discarded and protein was eluted with 150 µl native elution buffer (native binding buffer supplemented with 250 mM imidazole, pH 8.0). Extracts were loaded into Slide-A-Lyzer Mini Dialysis Units (10,000 MWCO, Pierce, Rockford, IL) and dialyzed overnight into Buffer D (20 mM HEPES-KOH pH 8.0, 20% glycerol, 100 mM KCl and 0.2 mM EDTA pH 7.9). Purified proteins were examined by Western blotting using PTB or His-tag specific antibodies.

### Phosphatase treatment and PKA assay

Uniformly-labeled [^32^P]UTP and [^32^P]CTP fosB RNA substrates containing the 3′ end of I4 were UV cross-linked and RNase A treated as described above. Six microliters of purified His-tagged PTB (0.25 µg/µl) was used in the cross-linking assays. For phosphatase treatment, protein was pre-incubated for 15 min with NE Buffer 3 (50 mM Tris-HCl pH 7.9, 100 mM NaCl, 10 mM MgCl_2_ and 1 mM DTT) followed by a 50 min incubation at 37°C with 3 µl (10 U/µl) of calf intestinal alkaline phosphatase (CIP, New England Biolabs, Ipswitch, MA). For the PKA phosphorylation reaction, protein was incubated with 3 µl of the catalytic subunit of cAMP dependent protein kinase (New England Biolabs, 2,500 U/µl), 200 µM ATP, 1× PKA Reaction Buffer (50 mM Tris-HCl pH 7.5 and 10 mM MgCl_2_) in a total reaction volume of 30 µl, for 1 hr at 30°C. Some of the His-tagged protein samples were phosphatase and PKA treated. In these instances, the phosphatase step was performed while the protein was still on the nickel beads, followed by wash out of the phosphatase, elution of the His-tagged protein and subsequent PKA treatment.

### Western blot analysis

Samples were mixed with an equal volume of 2× SDS sample buffer (125 mM TrisHCl pH 6.8, 20% glycerol, 4% SDS, 0.2% 2-mercaptoethanol (2-ME) and 0.001% bromphenol blue), boiled for 3 min and resolved by electrophoresis in an 8% SDS-polyacrylamide gel. Proteins were transferred to PVDF-Plus transfer membranes (Fisher Scientific, Pittsburgh, PA) for immunoblotting, using a semi-dry transfer apparatus (Owl Scientific, Woburn, MA) according to manufacturer's instructions.

PVDF membranes were then rinsed three times in 0.1% Tween-20 in TBS (TBS-T), blocked with 2% milk for 1 hr at room temperature, incubated with either PTB antibody (1/4,000 dilution; a gift of Dr. D.L. Black, UCLA) overnight at 4°C, washed with TBS-T and incubated for 60 min at room temperature with ECL peroxidase-labelled anti-mouse IgG (1/4,000; Amersham Biosciences). After three final washes in TBS-T, membranes were rinsed in TBS, immersed in chemiluminescence detection reagent (ECL Plus, Amersham Biosciences) and exposed to HyperFilm ECL film (Amersham Biosciences). Antibodies were stripped from the membrane by incubating in 62.5 mM TrisHCl (pH 6.8), 2% SDS, 100 mM 2-ME at 50°C for 30 min. Blots were then probed with an α-polyhistidine antibody (1/3,000 dilution; Clone His 1, Sigma-Aldrich). Images were captured using Kodak 1D Image Analysis Software.

### PTB depletion

Transcripts which have been shown to effectively deplete PTB from HeLa nuclear extracts [53] were generated from a plasmid containing the CUCUU-octamer sequence (a gift of Dr. C.W. Smith, University of Cambridge). RNA transcription was carried out in the presence of 100 µM biotin-14-CTP as previously described [53]. The biotinylated RNA was then bound to streptavidin magnetic beads (Invitrogen; 100 pmol of RNA/50 µl of beads) in 2× BW buffer (10 mM TrisHCl pH 7.5, 1 mM EDTA, 2 M NaCl). One hundred microliters of HeLa nuclear extract (2.6 µg/µl) was preincubated with 0.5 µl of 100 mM DTT and 34 U of RNasin for 15 min at room temperature, followed by incubation with the RNA-streptavidin beads for 30 min, using 1,440 fmol of RNA/µl of extract. The beads were removed from the extract using a magnet as described by the manufacturer.
